# Predictive performances of ALS and BLS termination of resuscitation rules in out-of-hospital cardiac arrest for different resuscitation protocols

**DOI:** 10.1186/s12873-022-00606-8

**Published:** 2022-03-27

**Authors:** Yu-Yuan Lin, Yin-Yu Lai, Hung-Chieh Chang, Chien-Hsin Lu, Po-Wei Chiu, Yuh-Shin Kuo, Shao-Peng Huang, Ying-Hsin Chang, Chih-Hao Lin

**Affiliations:** 1 Department of Emergency Medicine, National Cheng Kung University Hospital, College of Medicine, National Cheng Kung University, Tainan, 70403 Taiwan; 2 Department of Neurology, National Cheng Kung University Hospital, College of Medicine, National Cheng Kung University, Tainan, 70403 Taiwan

**Keywords:** Out-of-hospital cardiac arrest, Predictive performance, Termination of resuscitation, Resuscitation, Basic life support, Advanced life support

## Abstract

**Background:**

Resuscitation guidance has advanced; however, the predictive performance of the termination of resuscitation (TOR) rule has not been validated for different resuscitation protocols published by the American Heart Association (AHA).

**Methods:**

A retrospective study validating the basic life support (BLS) and advanced life support (ALS) TOR rules was conducted using an Utstein-style database in Tainan city, Taiwan. Adult patients with nontraumatic out-of-hospital cardiac arrests from January 1, 2015, to December 31, 2015, (using the AHA 2010 resuscitation protocol) and from January 1, 2020, to December 31, 2020, (using the AHA 2015 resuscitation protocol) were included. The characteristics of rule performance were calculated, including sensitivity, specificity, positive predictive value (PPV) and negative predictive value.

**Results:**

Among 1260 eligible OHCA patients in 2015, 757 met the BLS TOR rule and 124 met the ALS TOR rule. The specificity and PPV for predicting unfavorable neurological outcomes were 61.1% and 99.0%, respectively, for the BLS TOR rule and 93.8% and 99.2%, respectively, for the ALS TOR rule. A total of 970 OHCA patients were enrolled in 2020, of whom 438 met the BLS TOR rule and 104 met the ALS TOR rule. The specificity and PPV for predicting unfavorable neurological outcomes were 85.7% and 100%, respectively, for the BLS TOR rule and 99.5% and 100%, respectively, for the ALS TOR rule.

**Conclusions:**

Both the BLS and ALS TOR rules performed better when using the 2015 AHA resuscitation protocols compared to the 2010 protocols, with increased PPVs and decreased false-positive rates in predicting survival to discharge and good neurological outcomes at discharge. The BLS and ALS TOR rules can perform differently while the resuscitation protocols are updated. As the concepts and practices of resuscitation progress, the BLS and ALS TOR rules should be evaluated and validated accordingly.

## Background

Out-of-hospital cardiac arrest (OHCA) is a major global public health issue. The incidence of emergency medical services (EMS)-treated OHCA ranged from 30.0 to 97.1 per 100,000 person-years [1]. The rate of survival to discharge was generally poor and varied substantially, with a range from 3.1% to 20.4% [1]. Only 1% of patients with OHCA achieve good neurological outcomes after up to 37 min of cardiopulmonary resuscitation [2]. Eliastam et al. reported the futility of transporting certain patients who were unresponsive to resuscitation and was the first to mention the topic of termination of resuscitation (TOR) in the emergency department (ED) [3]. To decrease unnecessary consumption of medical resources and preserve energy for potential demand of emergent medical treatment in the ED, field termination was suggested as the best means to prevent needless transportation [4]. Many novel TOR rules have been developed in recent decades [5–7]. However, the basic life support (BLS) TOR rule and the advanced life support (ALS) TOR rule remain the mainstream rules [8, 9].

The BLS TOR rule was developed in 2002, with inclusion criteria of the cardiac arrest being unwitnessed by EMS personnel, the absence of a shockable rhythm and the absence of return of spontaneous resuscitation (ROSC) before transport [8]. Verbeek et al. proposed that emergency medical technicians-basic (EMTs-Basic) and emergency medical technicians-intermediate (EMTs-intermediate) performing BLS efforts in the field, consisting of the BLS team, could terminate resuscitation if all three criteria of the BLS rule were met [8]. Other than BLS resuscitation with cardiopulmonary resuscitation (CPR) and defibrillation, emergency medical technician-paramedics (EMT-Paramedics) were able to perform ALS procedures, including tracheal intubation and drug administration. The clinical prediction rule for OHCA patients attended by EMT-Paramedics was derived as the ALS TOR rule in 2007 [9], consisting of the absence of witnessing by bystanders or EMS personnel, bystander CPR, shock delivered and ROSC before transport [9]. Several retrospective studies have externally validated the BLS and ALS rules in the past decade [10–12].

The criteria of both the BLS TOR rule and the ALS TOR rule have not been modified in recent years. However, research on resuscitation has continued to progress to improve survival rates and neurological outcomes, and the resuscitation guidelines have been updated accordingly [13, 14]. Compared with the 2010 American Hospital Association (AHA) guidelines for CPR and emergency cardiovascular care, the 2015 AHA Guidelines emphasize team resuscitation, extracorporeal CPR and high-quality CPR, including minimizing interruptions in compression and avoiding overventilation, and clarify the recommendations for chest compression depth between 5 and 6 cm, instead of above 5 cm, and chest compression rates of 100 to 120 per minute, rather than above 100 per minute [14–16].

We suspected that the different CPR protocols could affect the predictive performance of the TOR rules. The aim of this study was to validate the BLS and ALS TOR rules in terms of the 2010 and 2015 AHA resuscitation guidelines.

## Methods

### Study design

This was a retrospective study using the Utstein-style population database of OHCA patients treated in Tainan city in 2015 and 2020. The 2010 AHA guidelines, which were published in November 2010, were adopted in Tainan city in January 2011 and have been fully implemented since December 2011. The 2015 AHA guidelines, which were published in October 2015, were adopted in Tainan city in January 2016 and have been fully implemented since December 2016. Considering the implementation of the educational program of CPR protocols and the optimization of the EMS system, this study validated the TOR rules for the 2010 resuscitation guidelines using the data between 1 January 2015 and 31 December 2015, while the TOR validation for the 2015 resuscitation guidelines used the data between 1 January 2020 and 31 December 2020. There was no community outbreak of COVID-19 in Tainan city during the study period and the EMS protocols were not modified in 2020.

### ALS and BLS teams

Tainan city has nearly 1.9 million residents living across an area of 2,192 km^2^. The National Fire Agency of Taiwan supervises the Fire Bureau of the Tainan City Government, which operates the EMS system in Tainan. There is one dispatch center consisting of experienced EMTs and nurses. EMTs are classified into three levels: EMT-Basic, EMT-Intermediate and EMT-Paramedic [17]. EMT-Intermediates can perform placement of advanced airways with laryngeal mask airways, while EMT-Paramedics can provide ALS treatments, including tracheal intubation and epinephrine use. The ALS team is defined as an EMS team with at least one EMT-Paramedic. Compared with the BLS team, the ALS team has an additional capability of endotracheal intubation and administration of intravenous epinephrine due to the involvement of EMT-Paramedic [17]. If cardiac arrest is recognized in emergency calls, the ALS team, instead of the team nearest to the scene, is dispatched.

All EMTs were certified to perform CPR according to the Taiwan guidelines at that time. The guidelines in Taiwan were modified by medical directors based on the AHA guidelines. The rate and depth of chest compression were consistent with the latest AHA guidance. EMTs performed CPR on scene for 4 min along with AED use and then transported the patients to the nearest hospitals with ongoing CPR in the ambulance. The corresponding educational training courses were designed for EMTs after the resuscitation guidelines were updated. There were no rules for prehospital TOR in the study city. All EMS-assessed patients with cardiac arrest received CPR unless do-not-attempt-resuscitation (DNAR) orders or obvious signs of irreversible death were present [18]. The signs of irreversible death included dependent lividity, rigor mortis, decomposition, decapitation, hemicorporectomy, and thermal carbonization without detectable vital signs [18]. Individuals with signs of irreversible death were generally not registered in the EMS data system.

There were 53 EMS stations in Tainan city. In 2015, there were 695 EMTs, which included 12 EMT-Basics, 555 EMT-Intermediates, and 128 EMT-Paramedics. In 2020, the numbers of EMT-Basic, EMT-Intermediate and EMT-Paramedic were 9, 632, and 150, respectively.

### Data collection

This study included all nontraumatic OHCA adult (18 years or older) patients who were transported by EMS personnel. Patients with a traumatic etiology, apparent death, existing DNAR orders, or known pregnancy were excluded. Those who had missing data regarding BLS/ALS TOR criteria or neurological outcomes were also excluded.

Demographic and clinical data were obtained from the database, including age, sex, location of cardiac arrest, response time, scene time, transport time, initial rhythm, airway management, use of epinephrine, the type of EMS team, bystander CPR, characteristic of witnesses, defibrillation, ROSC before transport and neurological outcomes.

The primary outcome measures were favorable neurological outcomes at discharge, defined as a Cerebral Performance Category (CPC) of 1 or 2 [19, 20]. The secondary outcome measure was survival to discharge.

### TOR rules

Those treated by the BLS or ALS teams were evaluated according to the BLS and ALS TOR rules, respectively. The BLS TOR rule recommends terminating resuscitation if all the following three criteria are met: the cardiac arrest was not witnessed by EMS personnel, no ROSC before transport, and no shock delivered before transport [8].

The ALS TOR rule recommends terminating resuscitation if all the following four criteria are fulfilled: the cardiac arrest was not witnessed, there was no bystander CPR, there was an absence of ROSC before transport, and an absence of defibrillation before transport [9].

### Statistical analysis

Categorical variables are summarized as numbers and percentages, while continuous variables are summarized as the means and standard deviations. Continuous variables were compared using two-sample independent t-tests. A two-tailed p value less than 0.05 was considered a statistically significant finding. Categorical variables were compared using chi-square tests. The test characteristics with corresponding 95% confidence intervals (CIs) were calculated, including sensitivity, specificity, false-positive rate (FPR), negative predictive value (NPV) and positive predictive value (PPV). The FPR (the probability that the patient survives when the rule suggests terminating resuscitation) and the PPV (the probability of death when the rule suggests terminating resuscitation) were among the more important tests. Statistical analyses were performed using IBM SPSS Statistics software (version 25.0; SPSS Inc., Chicago, IL, USA).

## Results

### Patient characteristics

**Fig. 1:**
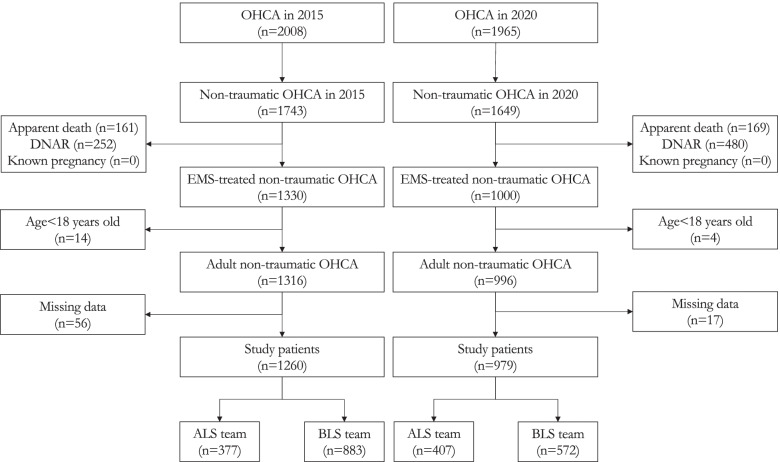
The study flow diagram. OHCA: out-of-hospital cardiac arrest, DNAR do not attempt resuscitation, EMS: emergency medical services, ALS: advanced life support, BLS: basic life support

A total of 2008 OHCA patients were assessed by EMTs in Tainan from January 1, 2015, to December 31, 2015. Of whom, 748 patients were excluded due to traumatic etiology (*n* = 265), apparent death (*n* = 161), existing DNAR orders (*n* = 252), age (under 18 years old; *n* = 14) and missing data (*n* = 56) (Fig. 1). Therefore, 1260 OHCAs in 2015 were enrolled in the final analysis, as the Group of 2015.

Among the 1965 OHCA patients who were assessed by EMTs in Tainan from January 1, 2020, to December 31, 2020, 986 patients were excluded as follows: 316 traumatic OHCA patients, 169 patients with apparent death, 480 patients who signed DNAR forms, 4 patients who were younger than 18 years old and 17 patients with missing data (Fig. 1). A total of 979 OHCAs in 2020 were enrolled in the final analysis, as the Group of 2020. There were fewer patients with existing DNAR orders in 2015 than in 2020 (14.5% vs. 29.1%, *p* < 0.001).

### Process and outcomes

Table 1 displays the demographic and prehospital characteristics of the eligible patients. There were no significant differences regarding age (68.5 ± 16.6 vs. 67.7 ± 15.9 years, *p* = 0.280) and sex (37.9% vs. 35.8%, *p* = 0.327). The mean response time, scene time and transport time were 6.7, 10.7 and 7.4 min in the Group of 2015, respectively, compared with 7.2, 13.5 and 7.8 min in the Group of 2020. The response time and the scene time in the Group of 2020 were significantly longer than those in 2015 (both *p* < 0.001). The rates of bystander CPR and defibrillation before transport in 2020 were significantly higher than those in 2015 (both *p* < 0.001).

**Table 1:** The clinical and prehospital characteristics of the enrollees

Characteristics	The Group of 2015 (N=1260)	The Group of 2020 (N=979)	P value
Age (year), mean ± SD	68.5±16.6	67.7±15.9	0.280
Female sex, n (%)	477(37.9)	350(35.8)	0.327
Location of cardiac arrest, n			
Home	1035(82.1)	742(75.8)	<0.001
Public space	166(13.2)	187(19.1)	0.080
Other	59(4.7)	50(5.1)	0.718
EMS care interval, min			
Response time	6.7±3.3	7.2±2.8	<0.001
Scene time	10.7±6.4	13.5±4.7	<0.001
Transport time	7.4±5.4	7.8±5.1	0.06
Initial rhythm			
VF/VT	114(9.0)	95(9.7)	0.597
PEA	256(20.3)	198(20.2)	0.956
Asystole	672(53.3)	511(52.2)	0.590
Other or unknown	33(2.6)	42(4.3)	0.029
Not recorded	185(14.7)	133(13.6)	0.462
Advanced airway by EMS			
Laryngeal mask airway	1040(82.5)	709(72.4)	<0.001
Endotracheal intubation	2(0.2)	22(2.2)	<0.001
Epinephrine use by EMS	60(4.8)	180(18.4)	<0.001
Cardiac arrest witnessed by			
Bystander	691(54.8)	374(38.2)	<0.001
EMS/first responder	70(5.6)	68(6.9)	0.175
Defibrillation before transport	124(9.8)	150(15.3)	<0.001
Bystander CPR	356(28.3)	477(48.7)	lt;0.001

*SD* standard deviation, *EMS* emergency medical service, *VF* ventricular fibrillation, *VT* ventricular tachycardia, *PEA* pulseless electrical activity, *CPR* cardiopulmonary resuscitation

Definition: response time, from emergency calls to ambulance arrival at the scene; scene time, between ambulance arrival and departure from the scene; transport time, from scene departure to hospital arrival

The rate of prehospital ROSC in 2020 was significantly higher than that in 2015 (*p* = 0.012). There were no significant differences regarding survival to hospital admission, survival to hospital discharge, or favorable neurological outcomes at discharge (all *p* > 0.05) (Table 2).

**Table 2:** The outcomes of the enrollees

Characteristics	The Group of 2015 (*N* = 1260)	The Group of 2020 (*N* = 979)	*P* value
ROSC before transport	18(1.4)	29(3.0)	0.012
Survival until hospital admission	223(17.7)	155(15.8)	0.242
Survival to discharge	56 (4.4)	50 (5.1)	0.462
Favorable neurological outcomes	34 (2.7)	31(3.2)	0.512

*ROSC* Return Of Spontaneous Circulation

Among the 1260 OHCAs in 2015, 757 (60.1%) and 124 (9.8%) met the BLS and ALS TOR rules, respectively (Table 3). Among the 979 OHCAs in 2020, 438 (44.7%) and 104 (10.6%) met the BLS and ALS TOR rules, respectively.

**Table 3:** Type of EMS team providing treatment

	The Group of 2015 (*N* = 1260)	The Group of 2020 (*N* = 979)	*P* value
Treated by BLS team	883(70.1)	572(58.4)	< 0.001
Met BLS TOR criteria	757(60.1)	438(44.7)	
Treated by ALS team	377(29.9)	407(41.6)	< 0.001
Met ALS TOR criteria	124(9.8)	104(10.6)	

*EMS* Emergency Medical Service, *BLS* Basic Life Support, *ALS* Advanced Life Support, *TOR* Termination Of Resuscitation

### Predictive performance of the BLS TOR rules

In 2015, 883 OHCA patients were resuscitated by the BLS team, of which 757 cardiac arrests met the BLS TOR rule. The sensitivity, specificity, FPR, PPV, and NPV of the BLS TOR rule for predicting mortality were 87.2% (95% CI 84.7–89.3), 50.0% (95% CI 32.8–67.2), 50.0% (95% CI 32.8–67.2), 97.8% (95% CI 96.4–98.6) and 13.5% (95% CI 8.3–21.0), respectively (Table 4). Decreased FPR and increased PPV in predicting unfavorable neurological outcomes were observed in both 2015 and 2020. In addition, the diagnostic accuracy of the BLS TOR rule in 2020 was more precise in predicting both death and poor neurological outcomes. A total of 572 OHCA patients were treated by BLS teams in 2020, of which 438 cardiac arrests met the BLS TOR rule. The sensitivity, specificity, FPR, PPV, and NPV of the BLS TOR rule for predicting death were 78.8% (95% CI 75.0–82.1), 69.2% (95% CI 48.1–84.9), 30.8% (95% CI 15.1–51.9), 98.2% (95% CI 96.3–99.1) and 13.4% (95% CI 8.4–20.7), respectively.

**Table 4:** Predictive performances of the TOR rules for neurological outcomes at discharge

Characteristics	CPC 3–5	CPC 1–2	Sensitivity, % (95% CI)	Specificity, % (95% CI)	FPR, % (95% CI)	PPV, % (95% CI)	NPV, % (95% CI)
BLS TOR rule							
The Group of 2015	865	18	86.7 (84.2–88.9)	61.1 (36.1–81.7)	38.9 (18.3–63.9)	99.0 (98.0–99.6)	8.7 (4.7–15.4)
Met criteria	750	7					
Not met criteria	115	11					
The Group of 2020	558	14	78.1 (74.4–81.5)	85.7 (56.2–97.5)	14.3 (2.5–43.8)	99.5 (98.2–99.9)	9.0 (4.9–15.4)
Met criteria	436	2					
Not met criteria	122	12					
ALS TOR rule							
The Group of 2015	361	16	34.0 (29.2–39.2)	93.8 (67.7–99.7)	6.2 (0.3–32.3)	99.2 (94.9–100.0)	5.9 (3.5–9.8)
Met criteria	123	1					
Not met criteria	238	15					
The Group of 2020	390	17	26.7 (22.4–31.4)	100.0 (77.1–100.0)	0 (0.0–22.9)	100.0 (95.6–100.0)	5.6 (3.4–9.0)
Met criteria	104	0					
Not met criteria	286	17					

*TOR* Termination Of Resuscitation, *CPC* Cerebral Performance Category, *CI* Confidence Interval, *FPR* False-Positive Rate, *PPV* Positive Predictive Value, *NPV* Negative Predictive Value, *BLS* Basic Life Support, *ALS* Advanced Life Support

## Predictive performance of the ALS TOR rule

In 2015, 377 OHCA patients were resuscitated by the ALS team, of which 124 cardiac arrests met the ALS TOR rule. The sensitivity, specificity, FPR, PPV, and NPV of the ALS TOR rule for predicting mortality were 35.0% (95% CI 30.0–40.3), 90.3% (95% CI 73.1–97.5), 9.7% (95% CI 2.5–26.9), 97.6% (95% CI 92.6–99.4) and 11.1% (95% CI 7.6–15.8), respectively (Table 5). A total of 407 OHCA patients were treated by the ALS team in 2020, of which 104 cardiac arrests met the ALS TOR rule. The sensitivity, specificity, FPR, PPV, and NPV of the ALS TOR rule for predicting unfavorable neurological outcomes were 26.7% (95% CI 22.4–31.4), 100.0% (95% CI 77.0–100.0), 0% (95% CI 0.0–23.0), 100.0% (95% CI 95.6–100.0) and 5.6% (95% CI 3.4–9.0), respectively.

**Table 5:** Predictive performances of the TOR rules for survival to discharge

Characteristics	Death	Survival	Sensitivity, % (95% CI)	Specificity, % (95% CI)	FPR, % (95% CI)	PPV, % (95% CI)	NPV, % (95% CI)
BLS TOR rule							
The Group of 2015	851	32	86.7 (84.2–88.9)	40.6 (24.2–52.9)	59.4 (47.1–75.8)	97.5 (96.0–98.4)	10.3 (5.8–17.3)
Met criteria	738	19					
Not met criteria	113	13					
The Group of 2020	546	26	78.8 (75.0–82.1)	69.2 (48.1–84.9)	30.8 (15.1–51.9)	98.2 (96.3–99.1)	13.4 (8.4–20.7)
Met criteria	430	8					
Not met criteria	116	18					
ALS TOR rule							
The Group of 2015	353	24	34.6 (29.7–39.8)	91.7 (71.5–98.5)	8.3 (1.5–28.5)	98.4 (93.7–99.7)	8.7 (5.7–13.0)
Met criteria	122	2					
Not met criteria	231	22					
The Group of 2020	383	24	27.2 (22.8–32.0)	100.0 (82.8–100.0)	0 (0.0–17.2)	100.0 (95.6–100.0)	7.9 (5.2–11.7)
Met criteria	104	0					
Not met criteria	279	24					

*TOR* Termination Of Resuscitation, *CI* Confidence Interval, *FPR* False-Positive Rate, *PPV* Positive Predictive Value, *NPV* Negative Predictive Value, *BLS* Basic Life Support, *ALS* Advanced Life Support

## Discussion

To identify the impact of the changes in the resuscitation guidelines, this study retrospectively evaluated the BLS and ALS TOR rules in patients with OHCA in 2015 and 2020. The results of our study showed that both the BLS and ALS TOR rules performed better in 2020 than in 2015, with increased PPVs and decreased FPRs in predicting survival to discharge and good neurological outcomes at discharge.

The proportion of survival and favorable neurological outcomes of this study was similar to previous Asian studies [21, 22]. No significant difference in survival rates or good neurological outcomes was identified between 2015 and 2020. However, the rate of favorable neurological outcomes increased slightly in 2020. There are two possible reasons for the disparity. First, after the resuscitation guidelines were updated, high-quality CPR was promoted and advocated for, which may consequently improve the outcomes of OHCA patients [23]. Second, a recent retrospective study demonstrated that an increased EMT-Paramedic ratio of the resuscitation team was associated with better neurological outcomes [17]. The incremental number of EMT-Paramedics trained in recent years may be another possible reason for the improved neurological outcomes.

During the COVID-19 pandemic, the increased incidence of OHCA patients, along with a reduction in the survival rate, was demonstrated in a previous meta-analysis [24], where the accuracy of the TOR rule may be obscured due to the expected reduction in the survival rate for OHCA patients during the pandemic period. Since no OHCA patient had been identified with a COVID-19 infection in 2020 in Tainan city, the COVID-19 pandemic did not affect the outcome of this study.

The rate of bystander CPR increased in 2020. Since the 2015 AHA guidelines emphasized the role of dispatchers in the recognition of cardiac arrest and instruction for CPR [25], the dispatcher-assisted cardiopulmonary resuscitation (DA-CPR) program was initiated in Tainan [26]. Subsequently, the bystander CPR rate increased in 2020, which is consistent with previous studies [27]. However, the rate of prehospital defibrillation and ROSC may not be associated with the implementation of the DA-CPR training program [27]. The role of DA-CPR program in the implementation of the TOR rules deserves further investigation.

The PPV of the BLS TOR rule was similar in previous validation studies in Japan, Korea, North America, and North Taiwan [7, 10, 21, 22]. A high FPR of the BLS TOR rule was reported in this study, which was consistent with previous Asian studies [7, 21, 28]. International variations in clinical practice and governmental policies for OHCA patients were demonstrated in a previous study [29]. In Taiwan, all OHCA patients should be transported to the hospital except those with apparent death, existing DNAR orders or families who declined transportation in consideration of patient status. Since the TOR in the field was legally permitted in some western regions instead of non-western regions, a few of those individuals with cessation of resuscitation would probably survive when transported [30, 31]. As a result, the predictive performance of the TOR rules in some western regions may be overestimated.

Many individuals were not transported to the hospital due to an existing DNAR order. The prevalence of DNAR orders has increased in recent years due to public education and the promotion of hospice care [32]. Due to the increasing number of patients with DNAR orders, the number of OHCA patients treated by EMTs decreased in 2020. As a result, the diagnostic accuracy of the TOR rule may be an overestimation since a minority of these untreated patients may survive if transported.

Both the BLS TOR rule and the ALS TOR rule performed better in 2020 in terms of predictiveness of neurological outcomes and survival. There are several possible rationales for the disparity. Grunau et al. suggested that the diagnostic accuracy of the TOR rules increased with increasing time to TOR rule application [32]. The response time and the transport time were generally shorter in Asian cities than in Western cities [33]. Prolonged scene time was observed in 2020, which implied an extension of resuscitation in the field and delayed the application of the TOR rules. Subsequently, the diagnostic accuracy in 2020 may have decreased.

A higher rate of ROSC before transport was observed in 2020 than in 2015. Given the similar rate of ROSC between 2015 and 2020, the findings of this study may infer that the number of false-positive, which was advised with needless termination, would decrease in 2020. In this study, one patient who met the BLS TOR rule with a good neurological outcome had ROSC during transportation in 2015. In other words, the patient was classified as false-positive. In contrast, none of the patients who fulfilled the BLS TOR rule had ROSC during transportation in 2020. The 2015 AHA guideline emphasized high-quality CPR, including adequate rate and depth of chest compressions, full chest recoil and minimizing interruptions between compressions, and avoiding excessive ventilation [25]. Since high-quality CPR increased the rate of prehospital ROSC [34], the FPR of the TOR rules may have subsequently decreased.

### Limitations

This study had several limitations. First, since the field TOR rules were not yet adapted in Taiwan, this study could only be conducted retrospectively. The missing outcome of some patients who were transferred to another hospital was described as data loss in this study. Variations in the EMS system and geographic features could also affect the mortality rate, dispatch service characteristics and other core characteristics of patients with OHCA [1, 21, 33, 35, 36]. Some characteristics of OHCA patients in 2015 and 2020 were different, which included the ratio of public location, the response time, and the ratio of witness cardiac arrest. The concept of hospice care is well promoted in Taiwan these years and we suspected the avoidance of unnecessary resuscitation efforts may lead to the disparity. The patients that experienced cardiac arrests at home may utilize ambulance less and thus the number of OHCA at home was decreased. On the other hand, the public awareness of early CPR is also improved, resulting in an elevating rate of bystander CPR. The impact of disparity of patient characteristics among years should warrant further studies.

Second, the resuscitation guidelines in 2015 highlighted postcardiac arrest care with therapeutic temperature management, extracorporeal membrane oxygenation and percutaneous coronary intervention, which have been shown to improve neurological outcomes [16, 37]. Our study did not analyze those confounders due to data unavailability. With the advancement of resuscitation and postcardiac arrest care, the rate of survival and favorable neurological outcomes could increase in the future. Therefore, the PPVs of the TOR rules will also correspondingly decrease. In other words, the TOR rules need to evolve with the time and circumstances of the different stages. New rules are expected to be derived to meet the needs of individual countries. Third, Marsden et al. proposed a TOR rule with an exclusion criterion of hypothermia [38]. The SOS–KANTO 2012 Study Group found that all patients who met their TOR rule but had good neurological outcomes had severe hypothermia with body temperatures below 30 degrees Celsius [5]. However, this study did not perform relevant analysis since EMS protocols in Tainan did not include temperature measurements. Finally, prolonged transport time indicated extended CPR in a moving ambulance. The quality of CPR during transportation is generally considered inadequate. In this study, the transport time was longer than thirty minutes in some individuals who lived in rural areas. Performing high-quality CPR during transportation is difficult due to frequent interruptions [39]. However, this study did not analyze chest compression fraction or interruption time due to incomplete data.

## Conclusion

In terms of predicting survival to discharge and good neurological outcomes at discharge, our study results showed that both the BLS and ALS TOR rules performed better when using the 2015 AHA resuscitation protocols compared to the 2010 protocols. The BLS and ALS TOR rules can perform differently while the resuscitation protocols are updated. As the concepts and practices of resuscitation progress, the BLS and ALS TOR rules should be evaluated and validated accordingly.

## Data Availability

The datasets used and analyzed during the current study are available from the corresponding author on reasonable request.

## Abbreviations

ALS: Advanced life support
AHA: American Hospital Association
BLS: Basic life support
CPR: Cardiopulmonary resuscitation
CPC: Cerebral-Performance Category
CI: Confidence Intervals
DA-CPR: Dispatcher-assisted cardiopulmonary resuscitation
DNAR: Do not attempt resuscitation
ED: Emergency department
EMS: Emergency medical services
EMT: Emergency medical technician
EMT-Basic: Emergency medical technician-basic
EMT-Intermediate: Emergency medical technician-intermediate
EMT-Paramedics: Emergency medical technician-paramedics
FPR: False-positive rate
NPV: Negative predictive value
OHCA: Out-of-hospital cardiac arrest
PPV: Positive predictive value
ROSC: Return of spontaneous resuscitation
TOR: Termination of resuscitation
